# Integrated transcriptomic and metabolomic investigation of the genes and metabolites involved in swine follicular cyst formation

**DOI:** 10.3389/fvets.2023.1298132

**Published:** 2024-01-11

**Authors:** Jiage Dai, Mingyue Pang, Jiabao Cai, Yan Liu, Yusheng Qin

**Affiliations:** ^1^Institute of Animal Husbandry and Veterinary Medicine, Beijing Academy of Agriculture and Forestry Sciences, Beijing, China; ^2^College of Animal Sciences and Technology, China Agricultural University, Beijing, China; ^3^Animal Science and Technology College, Beijing University of Agriculture, Beijing, China; ^4^College of Life Sciences and Food Engineering, Hebei University of Engineering, Handan, China

**Keywords:** follicular cyst, theca interna cells, granulosa cells, transcriptome, metabolome, tumor 1

## Abstract

Follicular cysts are a common reproductive disorder in mammals that is usually caused by stress. However, the pathogenesis of follicular cysts in sows remains unclear. To provide new insights into the mechanisms of follicular cyst formation in pigs, we conducted a combined transcriptomic and metabolomic analysis on theca interna and mural granulosa cells of follicular cysts and mature follicles. We identified 2,533 up-regulated and 1,355 down-regulated genes in follicular cysts, compared with mature follicles. These differentially expressed genes were mainly found in signaling pathways related to tumor formation and cortisol synthesis and secretion as shown by Ingenuity Pathway Analysis, which predicted 4,362 upstream regulatory factors. The combined gene expression and pathway analysis identified the following genes as potential biomarkers for porcine follicular cysts: *cytochrome P450 family 2 subfamily C polypeptide 18, L-lactate dehydrogenase, carbamoyl-phosphate synthase, fibroblast growth factor 7, integrin binding sialoprotein, interleukin 23 receptor, prolactin receptor, epiregulin, interleukin 1 receptor type II, arginine vasopressin receptor 1A, fibroblast growth factor 10, claudin 7, G Protein Subunit Gamma 3, cholecystokinin B receptor and cytosolic phospholipase A2*. Metabolomics analysis found significant differences in 87 metabolites, which were enriched in unsaturated fatty acid biosynthesis, and sphingolipid signaling pathways. These results provide valuable information on the molecular mechanisms of follicular cyst formation, which may facilitate the development of new therapeutics to prevent and treat follicular cysts.

## 1 Introduction

Ovarian cysts are a common reproductive disorder that significantly affect the reproductive performance of sows (1), with an incidence of 2.4–40% (2, 3). Ovarian cysts can be divided into follicular cysts and luteal cysts and can be unilateral or bilateral (4). During the normal estrus cycle, follicle-stimulating hormone stimulates follicle growth and development, and surges of luteinizing hormone trigger the release of ova from mature follicles. If hormone secretion is disturbed, the follicle cannot mature or ovulate, resulting in the formation of cysts with a diameter larger than 2 cm. The number and duration of cysts vary from individual to individual (2). Follicular cysts in sows can cause abnormal estrus (5), decreased pregnancy rate (2) and prolonged delivery intervals, as well as other complications, such as increased risk of uterine infection (6).

Studies in sows have analyzed follicular cyst morphology and fluid composition and steroid hormone concentration (7), changes in ovarian cyst cytoskeletal protein expression (8), and the association between RBP4 gene polymorphisms and follicle cysts (9). Stress stimulates the hypothalamic–pituitary–adrenal axis, resulting in glucocorticoid secretion (10–13), which affects the normal development of follicles (14, 15). Moreover, elevated levels of cortisol, the main glucocorticoid in pigs, can cause formation of follicular cysts (15). Gene mutations or polymorphisms also affect the normal development of follicles and ovulation, increasing the risk of follicular cysts. Despite these data, our understanding of the pathogenesis of follicular cysts is still limited and, therefore, there is a lack of effective therapies to prevent or treat follicular cysts.

Granulosa cells and theca cells secrete steroid hormones in the follicle. Mural granulosa cells secrete estrogen to regulate the growth and development of follicles, and luteinizing hormone/choriogonadotropin receptor (LHCGR) expressed by them is the main regulator of ovulation. Although the theca cells of follicles cannot directly synthesize estrogen, androstenedione synthesized under the action of luteinizing hormone is the substrate for the synthesis of estrogen by granulosa cells. Meanwhile, the membrane cells support the development of follicles and maintain the survival of granulosa cells.

In the study of follicular development, focus is usually given to granulosa cells and oocytes, while fewer studies focus on the associated membrane cells and granulosa cells., To comprehensively understand the pathogenesis of follicular cysts, and in view of the important role of membrane cells and parietal granule cells in follicular development and ovulation, we conducted in-depth comparative sequencing in combination with transcriptomic and metabolomic analysis of theca interna and mural granulosa (TIMG) cells to explore the key molecular pathways and biological processes of follicular cyst formation. These data provide new insights for the prevention and treatment of follicular cysts.

## 2 Materials and methods

This study was conducted at the Beijing Academy of Agriculture and Forestry Sciences, and the use of animals was approved by the Ethical Committee of Beijing Academy of Agriculture and Forestry Sciences (SYXQ-2012-0034).

### 2.1 Sample collection

**Figure 1:**
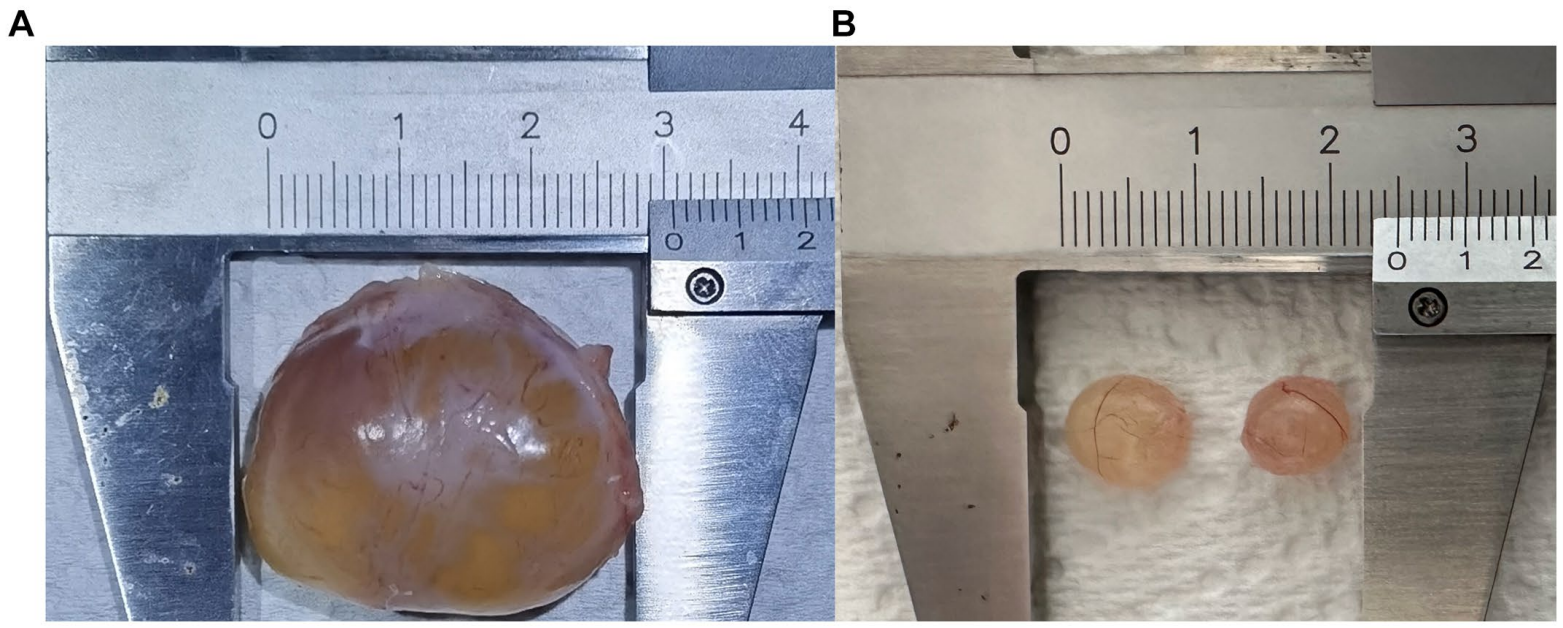
Follicular cyst **(A)** and mature follicles **(B)**.

The sows in this pig farm are mainly Landrace x Yorkshire sows. In large-scale pig farms around Beijing, most sows reach parity of 1–4, and some reach 5 or 6. The breeding standards for sows at the same stage of production are consistent. Professional and technical personnel used B-ultrasound (5 MHz, HS-1600 V, Honda, Japan) to identify follicle development status of sows. Follicular cysts and mature pre-ovulatory follicles were collected according to B-ultrasonography results (Figure 1). The collected follicles were transported to the laboratory within 2 h for further processing. The outer membrane of follicular cysts and mature follicles was separated as far as possible by ophthalmic tweezers, leaving a complete internal membrane enclosing the follicular fluid. After removal follicle fluid, the theca interna and mural granulosa complex was washed three times with PBS to wash away cell debris. The theca interna membrane and the mural granular cells were then collected and frozen in liquid nitrogen for transcriptome analysis. Transcriptomes were sequenced from three follicular cyst samples and three normal follicle samples, each from a different sow. Metabolomes were studied from six follicular cyst samples and six normal follicle samples, each from a different sow.

### 2.2 RNA extraction

Total RNA was extracted from follicular cysts and normal mature follicles using Trizol (MRC Corporation, the United States). RNA integrity and quantification were evaluated by 1% agarose gel electrophoresis and Agilent 2,100 assays.

### 2.3 Library preparation and sequencing

The library construction kit, NEB#7530 (#E7530, New England Biolabs), was used to enrich mRNA using Oligo (dT) magnetic beads. Purified mRNA was randomly sheared by ultrasound and the fragmented mRNA used as a template for first strand cDNA synthesis using random oligonucleotides as primers and the M-MuLV reverse transcriptase system. The RNA strand was then degraded by RNaseH, and the second cDNA strand was synthesized using the DNA polymerase I system. The cDNA library was purified and treated for terminal repair and joint sequencing, and the fragment size was selected and then amplified by PCR. Qubit2.0 and Agilent 2,100 assays were used to determine the concentration of the constructed library and the size of the inserted fragment. The qualified samples were sequenced using an Illumina-NovaSeq 6,000 sequencer and the paired-end read length was 150 bp. Fastp was used to perform quality control on raw reads and filter low-quality data to obtain clean reads. Clean reads were aligned to the ribosome database of the species using Bowtie 2. Reads on the aligned ribosome were removed without allowing mismatches, and the retained unmapped reads were used for subsequent transcriptome analysis. Based on the reference genome, the unmapped reads were aligned using HISAT2 software. Transcripts were reconstructed using Stringtie software and re-aligned to the reference genome, and RSEM was used to calculate the expression of all genes in each sample. In our analysis, we took the amount of expression in any two samples, calculated the Pearson correlation coefficient between these two samples, and then used these correlation coefficients to visually show the correlation between any two samples in the form of a heat map. The repeatability between samples within the group can be examined with this method.

The data that support the findings of this study have been deposited in the NCBI GEO datasets^1^ with accession number SUB13903899.

### 2.4 Metabolite extraction and detection

After samples were slowly thawed at 4°C, 50 ± l mg of each sample was added to pre-cooled methanol/acetonitrile/aqueous solution (2,2,1, v/v), mixed by vortexing, exposed to ultrasonic treatment at a low temperature for 30 min, left at −20°C for 10 min, centrifuged at 14000 g and 4°C for 20 min, and finally the supernatant was dried under vacuum. Then 100 μL acetonitrile solution (acetonitrile:water = 1:1, v/v) were added and the samples redissolved by swirling and centrifuged at 14000 g at 4°C for 15 min. Ten microliters of each sample were then used to prepare quality control samples, which were used to determine the state of the chromatography-mass spectrometry system before injection, and also to evaluate the stability of the system during the experiment.

The samples were separated by Agilent 1,290 Infinity LC ultra-high performance liquid chromatography with a hydrophilic interaction liquid chromatography (HILIC) column using the following parameters: column temperature 25°C; flow rate 0.5 mL/min; sample size 2 μL; mobile phase composition A: water +25 mM ammonium acetate +25 mM ammonia water, B: acetonitrile. The gradient elution procedure was as follows: 0–0.5 min, 95% B; 0.5–7 min, B changed linearly from 95 to 65%; 7–8 min, B changed linearly from 65 to 40%; 8–9 min, B maintained at 40%; 9–9.1 min, B changed linearly from 40 to 95%; 9.1–12 min, B maintained at 95%; The samples were placed in a 4°C automatic injector during the entire analysis process. To avoid influence from fluctuation of the instrument detection signal, the samples were analyzed continuously in random order. Quality control samples were inserted into the sample queue to monitor and evaluate the stability of the system and the reliability of the experimental data.

An AB Triple TOF 6600 mass spectrometer was used to collect primary and secondary spectra of samples. The electrospray ionization source conditions after HILIC chromatographic separation were: ion source gas1: 60, ion source gas2: 60, curtain gas: 30, source temperature: 600°C, IonSapary Voltage Floating ±5,500 V (positive and negative modes); time of flight mass spectrometry (TOF MS) scan m/z range: 60–1,000 Da, product ion scan m/z range: 25–1,000 Da, TOF MS scan accumulation time 0.20 s/spectra, product ion scan accumulation time 0.05 s/spectra. Secondary mass spectrometry was obtained using information dependent acquisition (IDA) and high sensitivity mode, declustering potential: ±60 V (positive and negative modes), collision energy: 35 ± 15 eV. IDA settings were as follows: exclude isotopes within 4 Da, candidate ions to monitor per cycle: 10.

Positive ion mode (POS) and negative ion mode (NEG) were used in combination to improve the metabolite coverage rate and detection effect. Peak identification, peak filtering and peak alignment were carried out on the original data to obtain mass-charge ratio, retention time and peak area data, and precursor molecules under positive and negative ion modes were obtained. To make comparisons between data of different orders of magnitude, quantitative results were normalized and then data identification and quantification were performed.

### 2.5 Identification of single-nucleotide polymorphisms

After quality control, mRNA sequencing data were compared with the pig reference genome (Sscrofa11.1) and sorted according to chromosomal coordinates. GATK software was used to detect potential SNP loci. The SNPs related to differentially expressed genes (DEGs) were analyzed to help determine the genes related to the occurrence of luteal cysts.

### 2.6 Analysis of DEGs

DESeq2 software was used for differential expression analysis. The Benjamini-Hochberg correction method was used to correct the significance *p*-value obtained from the original hypothesis test and the error discovery rate (FDR) was obtained. Will | log2 (Fold Chang) | > l and FDR < 0.05 were used as the criteria for differential expression gene screening. The selected DEGs were mapped into each term/pathway in the Gene Ontology (GO) and Kyoko Encyclopedia of Genes and Genomes (KEGG) databases, the number of genes that could be enriched in each term/pathway was calculated, and the GO terms and KEGG pathways with significant enrichment of differential genes were identified. To predict the major upstream regulators of cyst follicles by all DEGs, we used Ingenuity Pathway Analysis (IPA, version101138820, Ingenuity Systems, Mountain View, CA). This analysis assumes a difference in downstream genes by computing an overlap value of p and an activation z-score to provide a more reliable prediction.

### 2.7 Metabolite identification and differential metabolite abundance analysis

The raw file was imported into the Community Detection (CD) Library search software and the retention time, mass-charge ratio and other parameters were simply screened, and then the peak alignment of different samples was performed according to the retention time deviation of 0.2 min and the mass deviation of 5 ppm so as to make the identification more accurate. Then the peak was extracted according to the set mass deviation of 5 ppm, signal strength deviation of 30%, signal-to-noise ratio of 3, minimum signal strength of 100,000, plus ion and other information. Meanwhile, the peak area was quantified, and then the target ion was integrated. The molecular formula was predicted by molecular ion peak and fragment ion, and compared with mzCloud,^2^ mzVault and Masslist databases. Background ions were removed with blank samples, and the quantitative results were normalized to obtain final identification and quantitative results. Orthogonal partial least squares discriminant analysis (OPLS-DA) was used to establish the discriminant model. According to the projected value in the model [Variable Importance in Projection (VIP) = 1.00], differential metabolites were extracted. The hypothesis test value (*p*-value) of potential differential metabolites between the two groups was obtained by Student’s t-test, and the fold-change value of metabolites was calculated by comparing the average value of each peak area.

### 2.8 Enrichment analysis and topological analysis of metabolite pathways

The comprehensive analysis of differential metabolite pathways included enrichment analysis and topological analysis. The National Institute of Standards and Technology,^3^ Chemical Entities of Interest,^4^ and KEGG^5^ online databases were used to identify metabolites, investigate biological functions and build pathways. Metabo Analyst 5.0 software^6^ was used to analyze different metabolites identified by KEGG pathway analysis. The degree of centrality or the relative betweenness centrality measure was used to determine the importance of the pathway in the network diagrams. The hypergeometric test or Fisher’s exact test was used to determine the significance of the pathway.

### 2.9 Verification of DEGs by RT-qPCR

Primers were synthesized by Shanghai Bioengineering Co., Ltd. (Shanghai, China) and their sequences are presented in Supplementary file 1. The iScript kit (Bio-Rad Laboratories, Hercules, CA, United States) and the Bio-Rad Chrome real-time qPCR system were used to perform qPCRs. PCRs consisted of 5 μL SYBR green premix, 0.3 μL forward primer, 0.3 μL reverse primer, 4 μL cDNA, and 0.4 μL dH_2_O. The reaction program was 95°C 120 s 1 cycle, 95°C 10 s, 60°C 30 s, 72°C 25 s, 40 cycles, and 72°C 420 s 1 cycle. Three technical replicates and three biological replicates were performed for each group of samples and the relative expression level of each gene was calculated using the 2^−ΔΔCT^ method (16).

## 3 Results

### 3.1 Transcriptome sequencing

**Figure 2:**
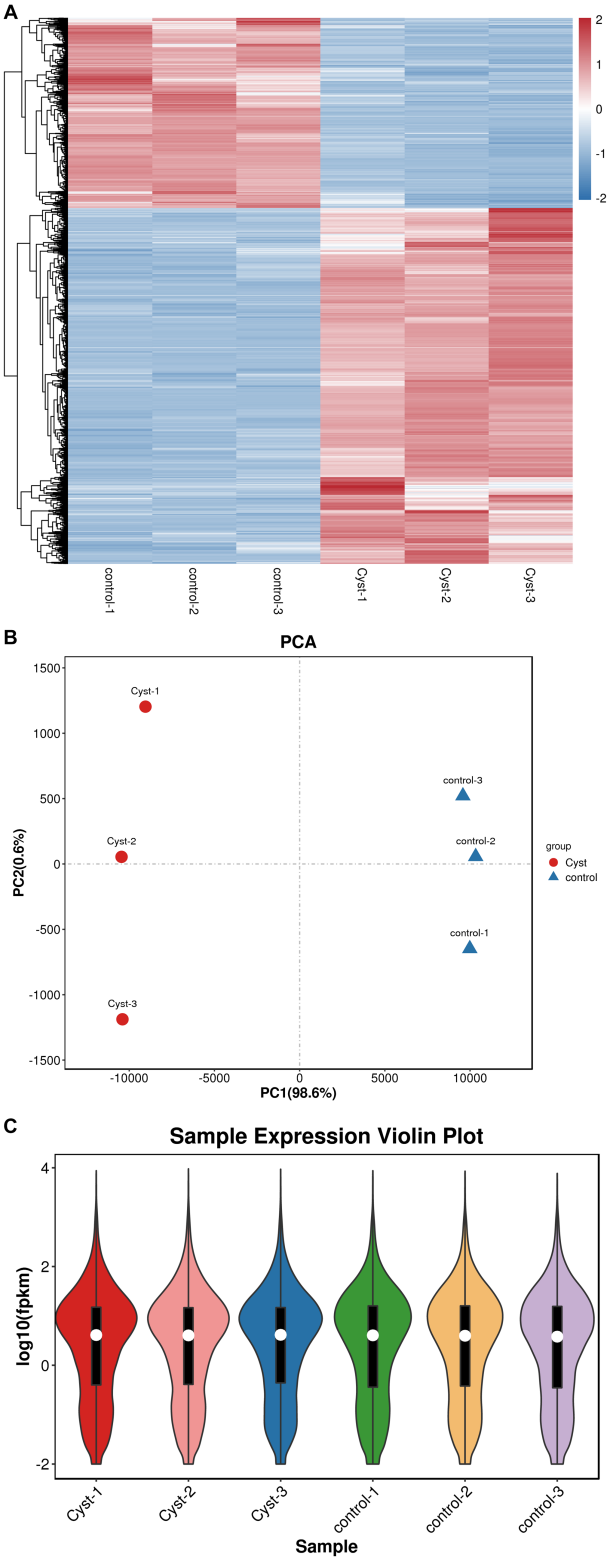
Quality control and comparison of results. **(A)** Cluster heatmap analysis of mature follicular membrane and follicular cyst membrane. **(B)** PCA analysis. The x-axis shows PC1 (98.6%) and the y-axis shows PC2 (0.6%). Red, cyst; blue, control. **(C)** Expression violin plot of average expression distribution in cystic and normal samples. The x-axis shows sample names and the y-axis shows log10 (fpkm).

The transcriptomes of follicular cysts and normal follicular theca interna were analyzed to explore the pathogenesis of follicular cysts. Samples were sequenced using the Illumina HiSeq2500 sequencing platform. After quality control of the original data, approximately 5 GB of clean reads were obtained for each sample. The Q20 value of the data, tested by Fast QC, was higher than 95% for all samples, and the proportion of low-quality bases was relatively low in all samples (Figure 2A), indicating that the sequencing quality was high. The high-quality clean reads from the sequencing data were compared with the reference genome using Hisat2 software. The comparison rate of all processing was above 95%, which met the requirements for subsequent gene expression analysis. More than 85% of the sequencing data were comparable with the exon regions of genes, and other reads were comparable with introns or inter-intronic regions, which further indicated the high quality of the sequencing data. Principle component analysis gave a PC1 value of 98.6% and a PC2 value of 12.8% (Figure 2B). The degree of separation of scatter points on PC1 and PC2 indicated significant differences between the cystic and normal samples, while the difference within samples was small. The distribution trend of gene expression was consistent between cystic and mature follicles, and the log10 (fpkm) value was concentrated between −2 and 4 (Figure 2C). All raw sequence data were deposited in the China National GeneBank DataBase (CNGBdb) Nucleotide Sequence Archive (CNSA)^7^ with accession number CNP0004553.

### 3.2 Identification and functional annotation of DEGs

**Figure 3:**
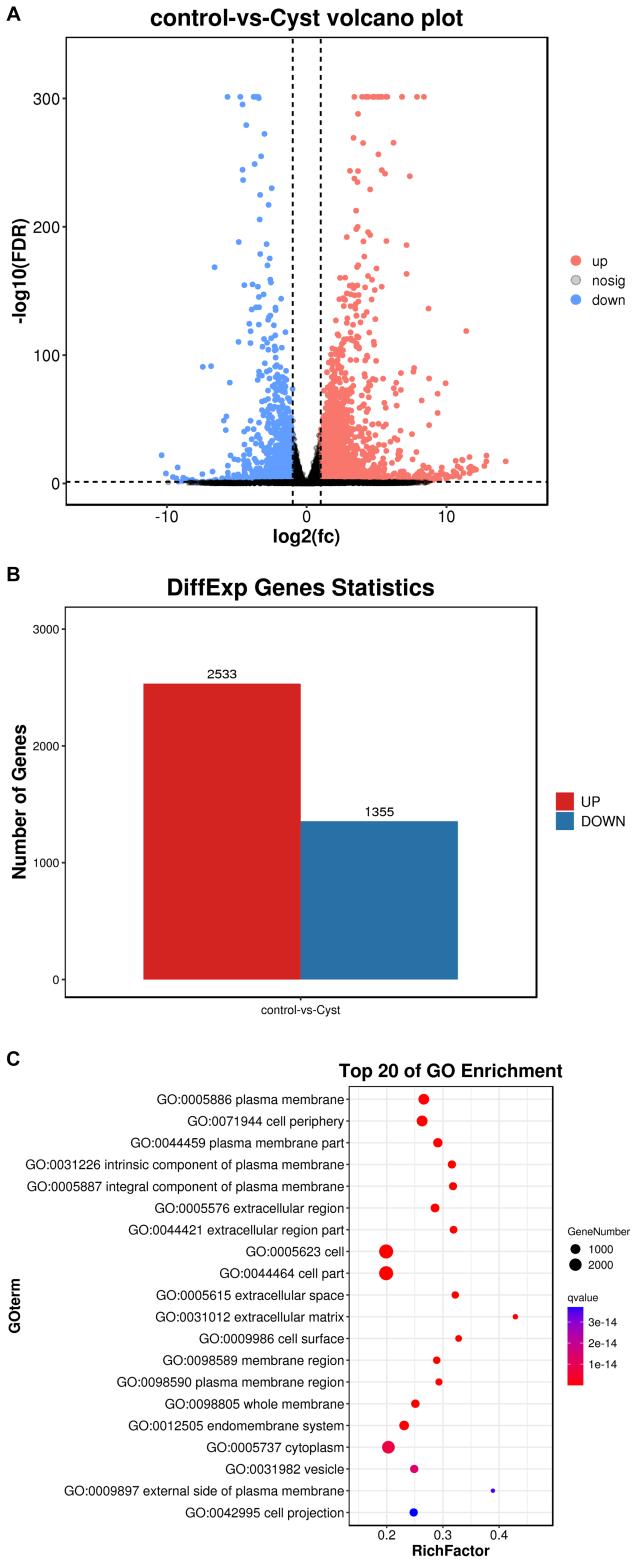
Comparison of DEGs in TIMG cells of follicular cysts. **(A)** Volcano plots of DEGs. The x-axis shows the log2(fc) of gene expression and the y-axis shows -log10(FDR). Red, up-regulated genes; blue, down-regulated genes. **(B)** Bar chart of DEGs. The x-axis shows control-vs-cyst representing sample grouping and the y-axis shows the number of genes. Red, up-regulated genes; blue, down-regulated genes. **(C)** GO enrichment analyses of Dimethyl sulfate(DMs). The ratio of the number of DMs to the total metabolite number is represented by the enrichment factor. Size of dots: number of metabolites; color of dots: range of *p*-values. *p* < 0.05.

In this study, we performed a volcano map analysis based on the significantly different genes in each comparison group (Figure 3A). It provides a visual representation of the differential genes between the comparison groups, and the closer the genes are to the two ends, the greater the degree of difference. We identified 3,888 DEGs in follicular cysts relative to mature follicles, including 2,533 significantly up-regulated genes and 1,355 significantly down-regulated genes.

To determine the function of DEGs, we performed GO and KEGG signaling pathway enrichment analysis. The results of GO cluster analysis showed that DEGs were significantly enriched in 1088 functional categories in the GO database (*p* < 10^−5^), including 918 biological processes, 88 cellular components and 83 molecular functions. The top 20 most significant functional categories are shown in Figure 3C. These categories include cell membrane-related biological processes, such as plasma membrane, cell periphery, plasma membrane part, cell adhesion, biological adhesion, single−multicellular organism process, system development, regulation of multicellular organismal process, intrinsic component of plasma membrane, integral component of plasma membrane, extracellular region, multicellular organismal process and extracellular region part.

**Figure 4:**
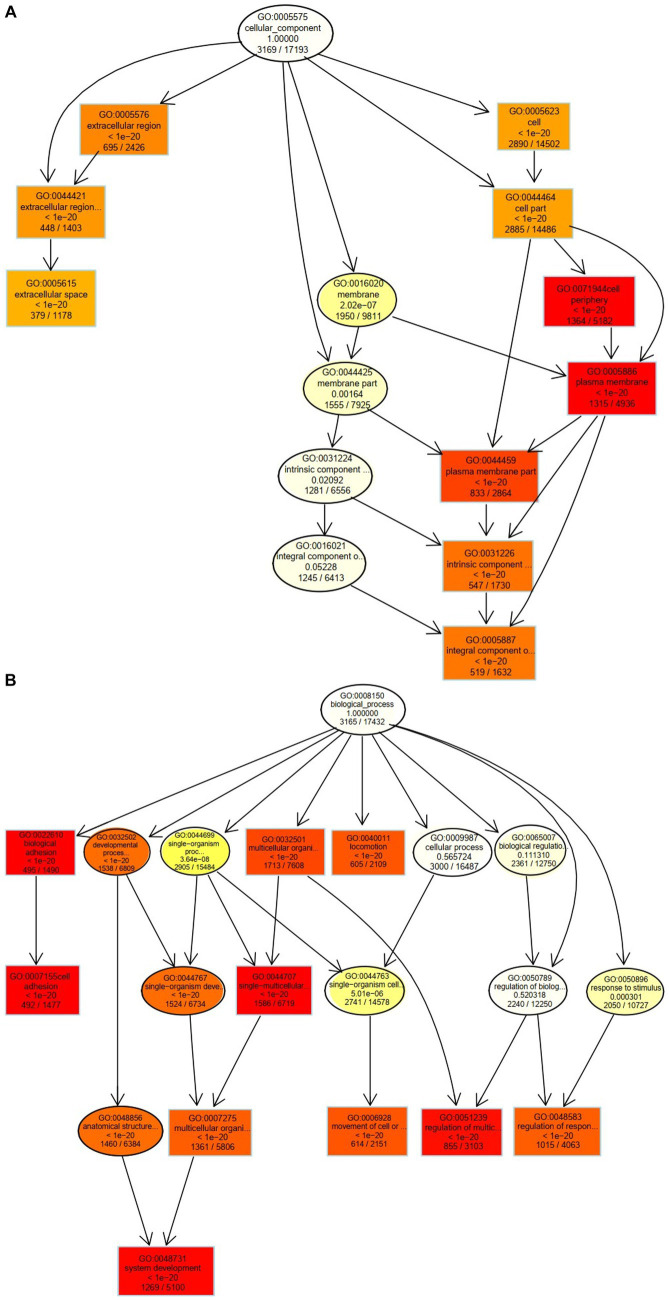
Cellular and biological processes altered in TIMG cells of follicular cysts. **(A)** Two major processes that affect the formation of follicular cysts. **(B)** DEGs are involved in the activation and regulation of multicellular organisms.

The 10 nodes with the highest enrichment were selected as the main nodes for a directed acyclic graph (DAG) and the hierarchical relationships of these nodes were analyzed. DAG results showed that the DEGs affected cyst formation by two main processes: activation of biological adhesion and promoting cell adhesion (Figure 4A). DEGs were also involved in activation and regulation of the multicellular organismal process (Figure 4B). These data indicate that these biological processes influence the development of follicular cysts through the cell periphery, plasma membrane and plasma membrane parts.

**Figure 5:**
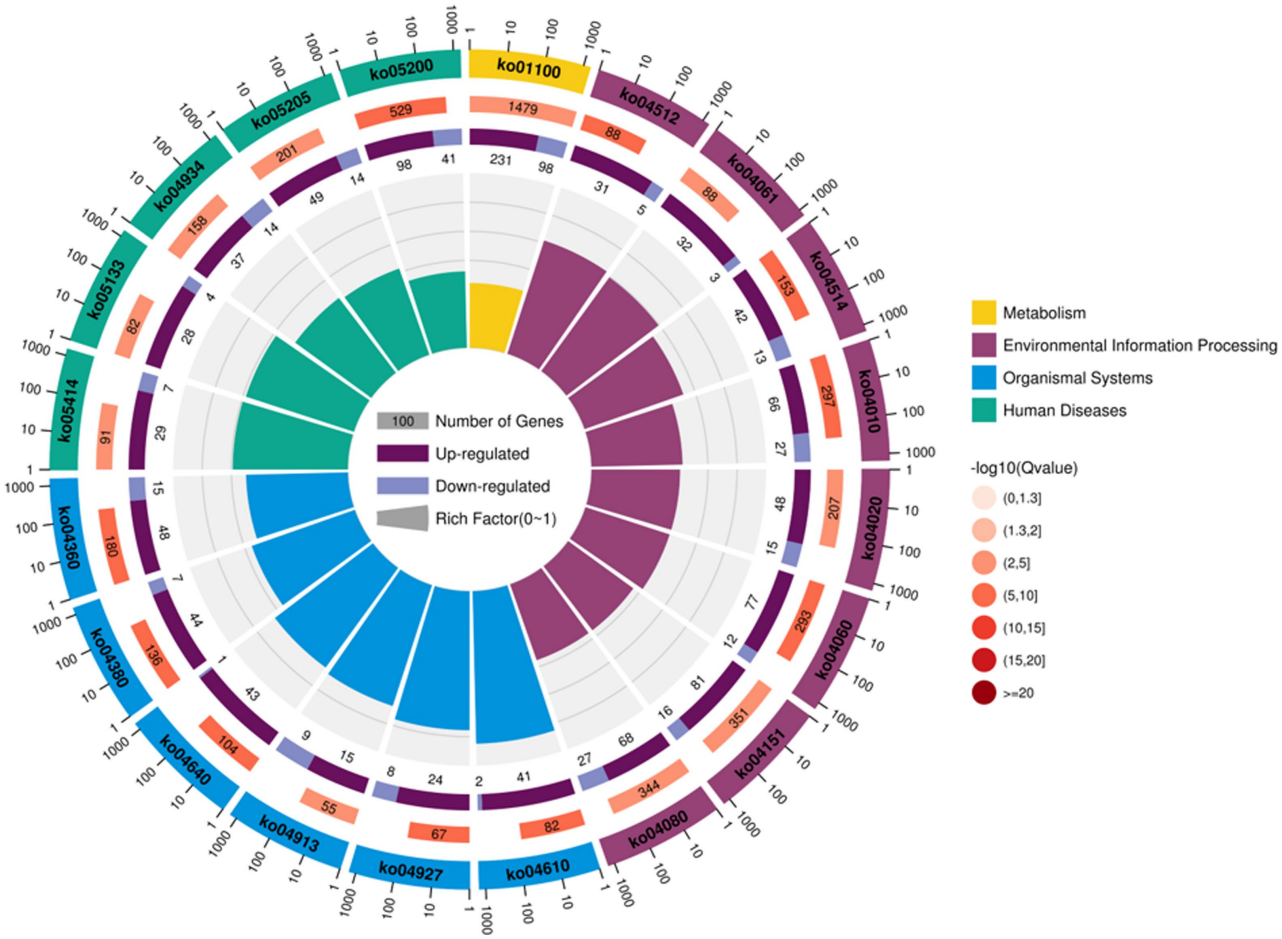
Annotation of KEGG functions of DEGs in the theca interna and mural granulosa cell complex.

For follicular cysts, KEGG enrichment analysis showed that the top 20 pathways were related to human diseases, cellular processes, metabolism, organismal systems, environmental information processing and genetics. In six categories, such as information processing, a total of 107 pathways reached the difference display level (Figure 5). Among the top 20 pathways, five were related to disease, most of which were related to cancer formation. These include pathways in cancer, proteoglycans in cancer and the mitogen-activated protein kinase (MAPK) signaling pathway. Other processes, including cortisol synthesis and secretion, cell adhesion molecules and cytokine-cytokine receptor interaction, are also involved in the formation of follicular cysts.

### 3.3 Screening of genes related to follicular cyst development based on transcriptomic results

For follicular cysts, 30 significantly enriched pathways (*p* < 10^−5^) were selected and parsed based on the fold-change of DEGs |log2(FC)| > 5 (Supplementary file 2). The pathways with the highest fold-change included metabolic pathways, neuroactive ligand-receptor interaction, MAPK signaling pathway, PI3K-Akt signaling pathway, ECM-receptor interaction, cytokine-cytokine receptor interaction, cell adhesion molecules, cortisol synthesis and secretion and calcium signaling pathway. Among them, MAPK signaling pathway, cortisol synthesis and secretion, cytokine−cytokine receptor interaction, cell adhesion molecules and the MAPK signaling pathway are within the top 20 pathways, indicating that these pathways are closely related to the development of follicular cysts. From the combined gene expression and pathway analysis data, we selected 15 genes related to follicular cyst detection that were KEGG validated: *cytochrome P450 family 2 subfamily C polypeptide 18 (CYP2C18), L-lactate dehydrogenase (LDHB), carbamoyl-phosphate synthase (CPS1), fibroblast growth factor 7 (FGF7), integrin binding sialoprotein (IBSP), interleukin 23 receptor (IL23R), prolactin receptor (PRLR), epiregulin (EREG), interleukin 1 receptor type II (IL1R2), arginine vasopressin receptor 1A (AVPR1A), fibroblast growth factor 10 (FGF10), claudin 7 (CLDN7), G Protein Subunit Gamma 3 (GNG3), cholecystokinin B receptor (CCKBR) and cytosolic phospholipase A2 (PLA2G4E)*.

### 3.4 Prediction of upstream transcription regulators

**Figure 6:**
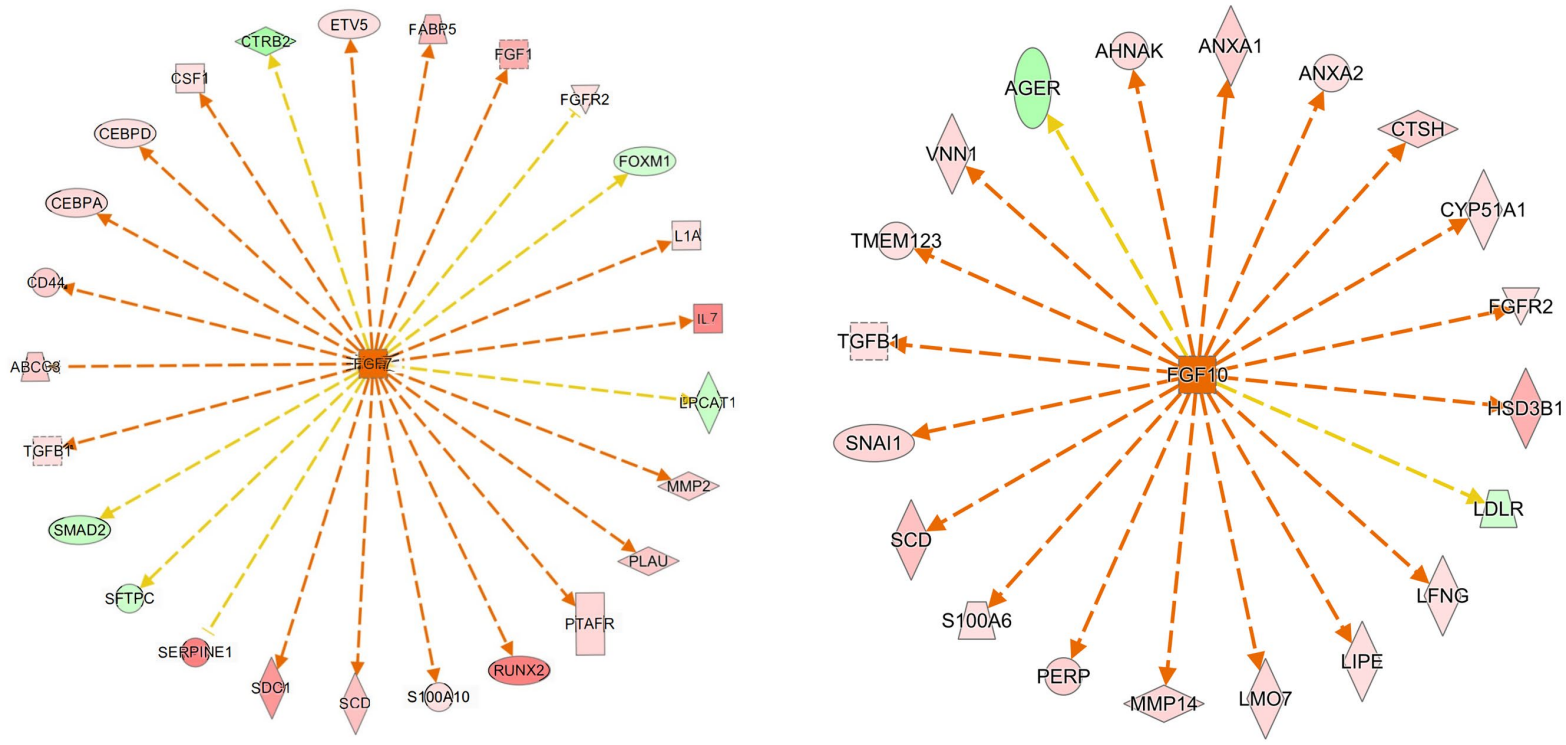
Prediction of upstream transcription regulators. An upstream regulatory network diagram shows the interactions between upstream regulators and their directly related downstream molecules that coexist in the dataset. The orange line indicates the expression state with consistent activation between the upstream regulator and the gene, and the yellow line indicates the expression state with inconsistent activation between the upstream regulator and the gene. In Newwork Shapes, the vertical diamond represents Enzyme, the horizontal diamond represents Peptidase, the circle represents Other, the oval represents Transcription, the positive trapezoid represents Transporter, the inverted trapezoid represents microRNA, the vertical rectangle represents G-protein Coupled Receptor, the inverted triangle represents Kinase, and the dotted square represents Growth Factor. The red in the graph represents increased measurement is more extreme in dataset. The pink in the graph represents increased measurement is less in dataset. The Dark green in the graph represents decreased measurement is more extreme in dataset. The Light green in the graph represents decreased measurement is less in dataset.

We used IPA analysis to predict 4,362 upstream regulatory factors (Supplementary file 3). FGF7 regulated the PPAR signaling pathway (*FABP5* and *SCD*) and the MAPK signaling pathway (*FGF1*, *FGFR2*, *IL1A*, *TGFB1*, and *CSF1*) and pathways in cancer (*IL7*, *CD44*, *SMAD2*, and *MMP2*) may be involved in the formation of follicular cysts (Figure 6A). The predicted regulatory factor FGF10 involved in the formation of follicular cysts through regulating the MAPK signaling pathway (*FGFR2*), and cortisol synthesis and secretion (*CYP51A1, HSD3B1*, and *LDLR*), biosynthesis of unsaturated fatty acids (*SCD*; Figure 6B).

### 3.5 Overview of metabolite detection and principal component analysis

**Figure 7:**
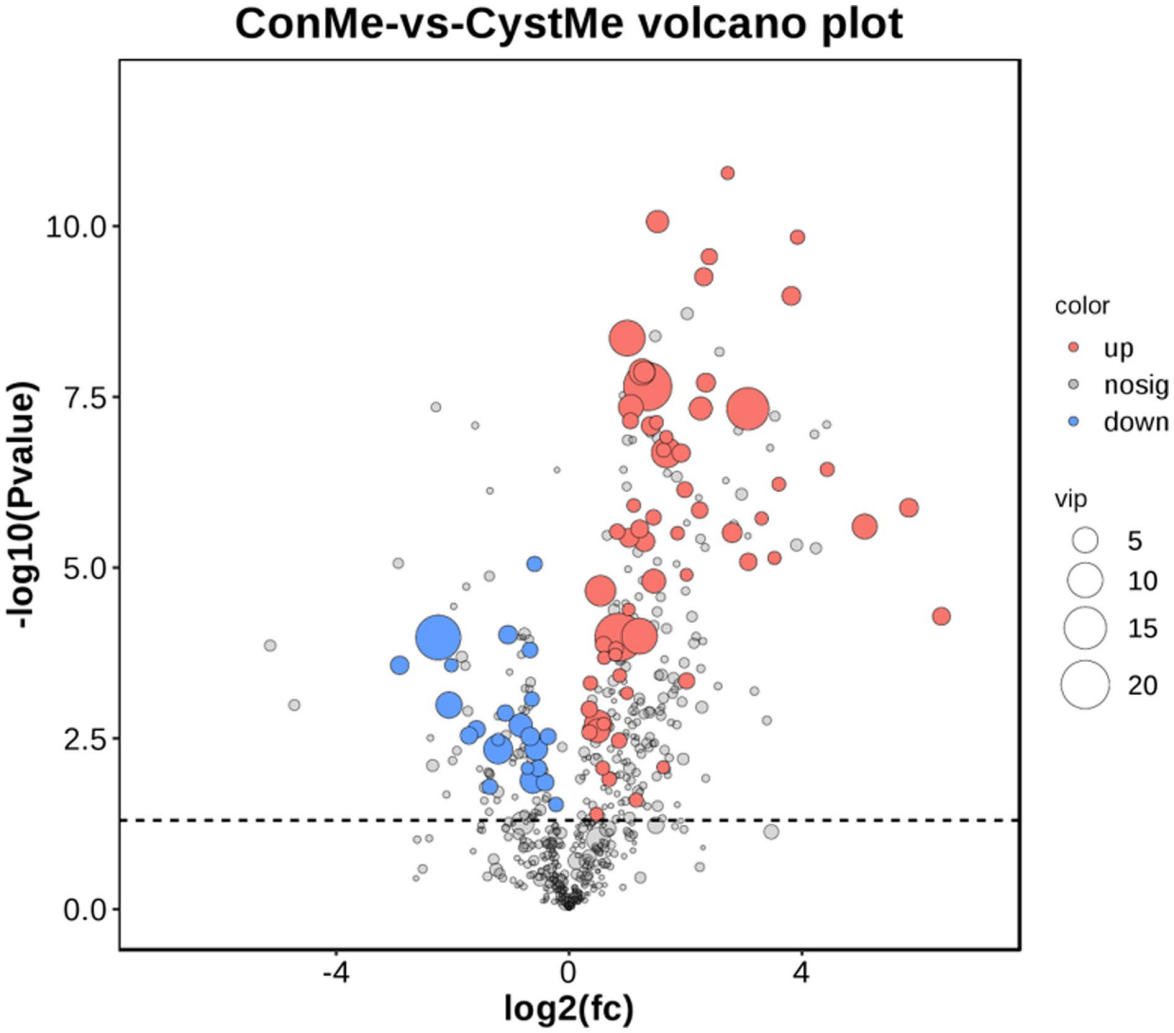
Differential metabolite volcano plot and number of metabolites. The x-axis shows the log2(fc) of gene expression and the y-axis shows -log10 (*p* value). Red, up-regulated metabolites; blue, down-regulated metabolites.

Gas Chromatography–Mass Spectrometry (GC/MS) was used to detect metabolites from the theca interna of follicular cysts and normal mature follicles. After quality control, a total of 9,226 original data points were obtained from follicular cysts and normal mature follicles, and 1,004 metabolites were annotated (Figure 7).

**Figure 8:**
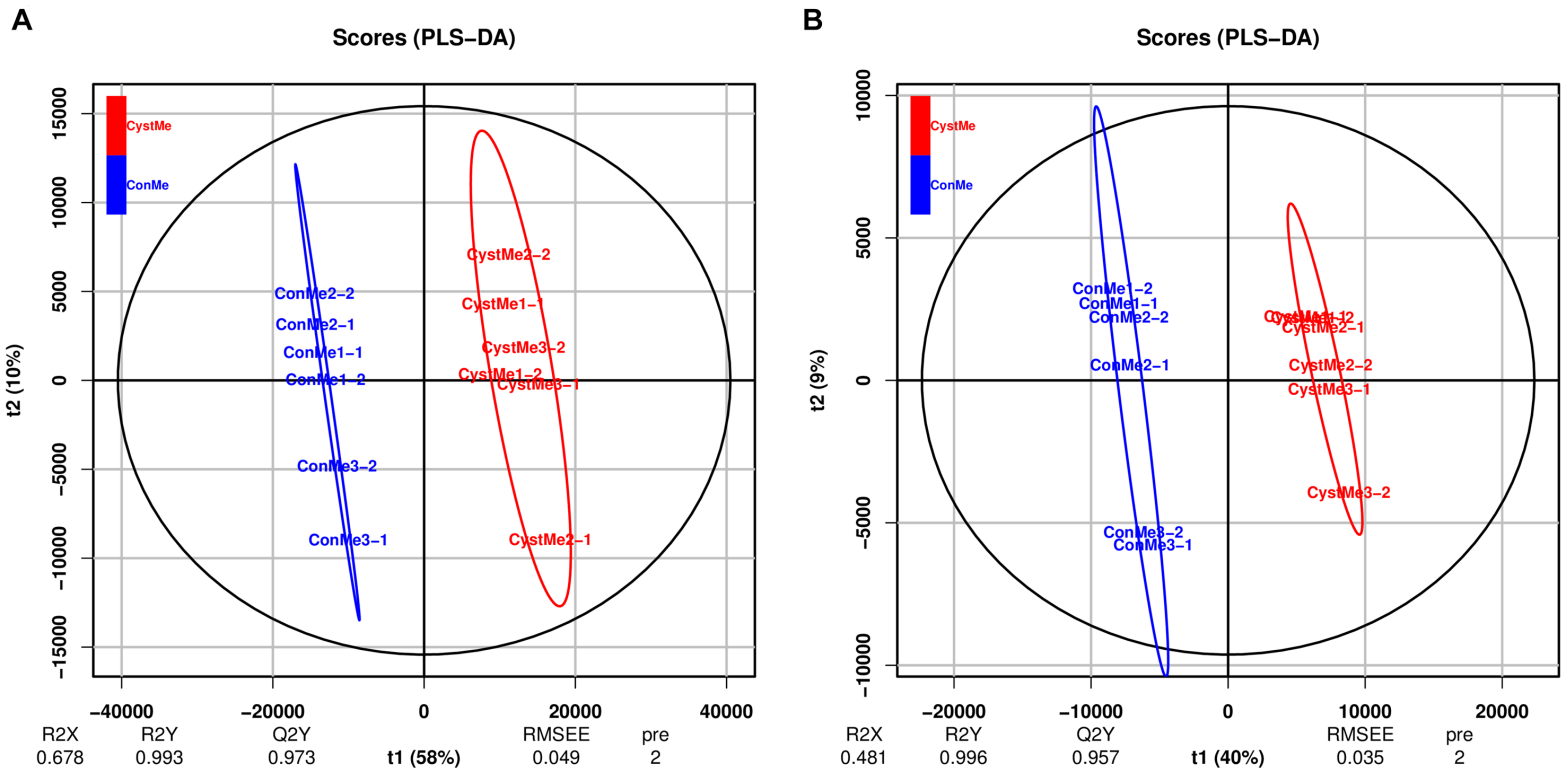
Partial least squares discriminant analysis of the theca interna and mural granulosa cell complex of follicular cysts and mature follicles. **(A)** Positive ion mode, POS. **(B)** Negative ion mode, NEG.

Principal component analysis showed differences in the metabolic profiles of different samples (Figure 8). The reliability and quality of the model was tested by seven-fold cross-validation. After 200 permutations of different treatments, different random R2 and Q2 values were obtained. R2Y and Q2Y were used to evaluate the validity of the model, and the orthogonal partial least squares discriminant analysis of the first principal component and the second principal component showed clear differences between cystic and mature follicles. Overall, different follicles have different metabolic patterns and therefore have different physiology.

### 3.6 KEGG annotation of differentially expressed metabolites

**Figure 9:**
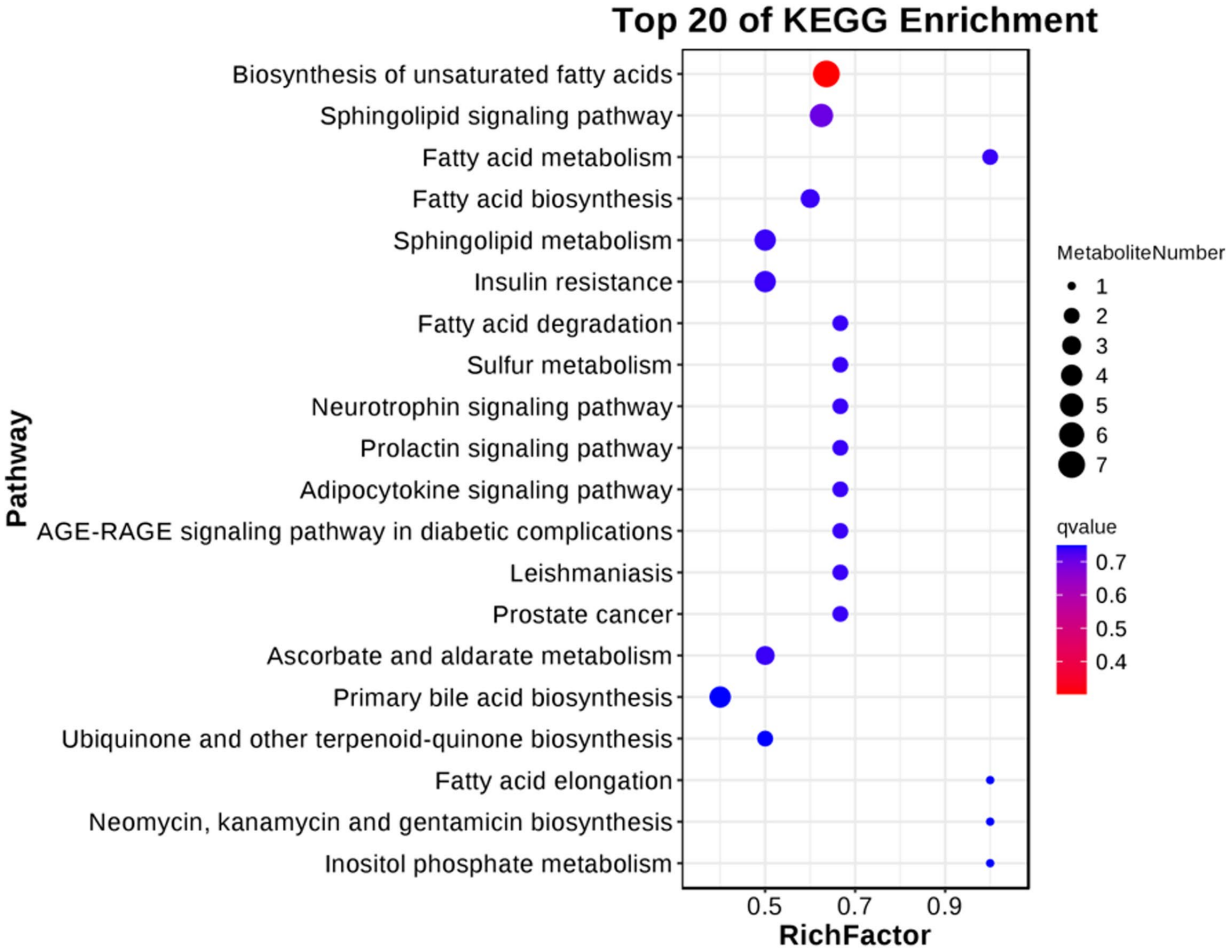
KEGG enriched bubble diagram of DEGs in the theca interna and mural granulosa cell complex of follicular cysts and mature follicles.

For follicular cysts, we classified the pathways enriched in differentially expressed metabolites (Figure 9). The top 20 pathways were related to cancer, genetic information processing and diseases. Further screening by *p*-value obtained two significantly enriched pathways (*p* < 0.05), unsaturated fatty acid biosynthesis and the sphingolipid signaling pathway, which indicates that these biological processes are closely related to the development of follicular cysts.

### 3.7 Validation by quantitative RT-PCR

**Figure 10:**
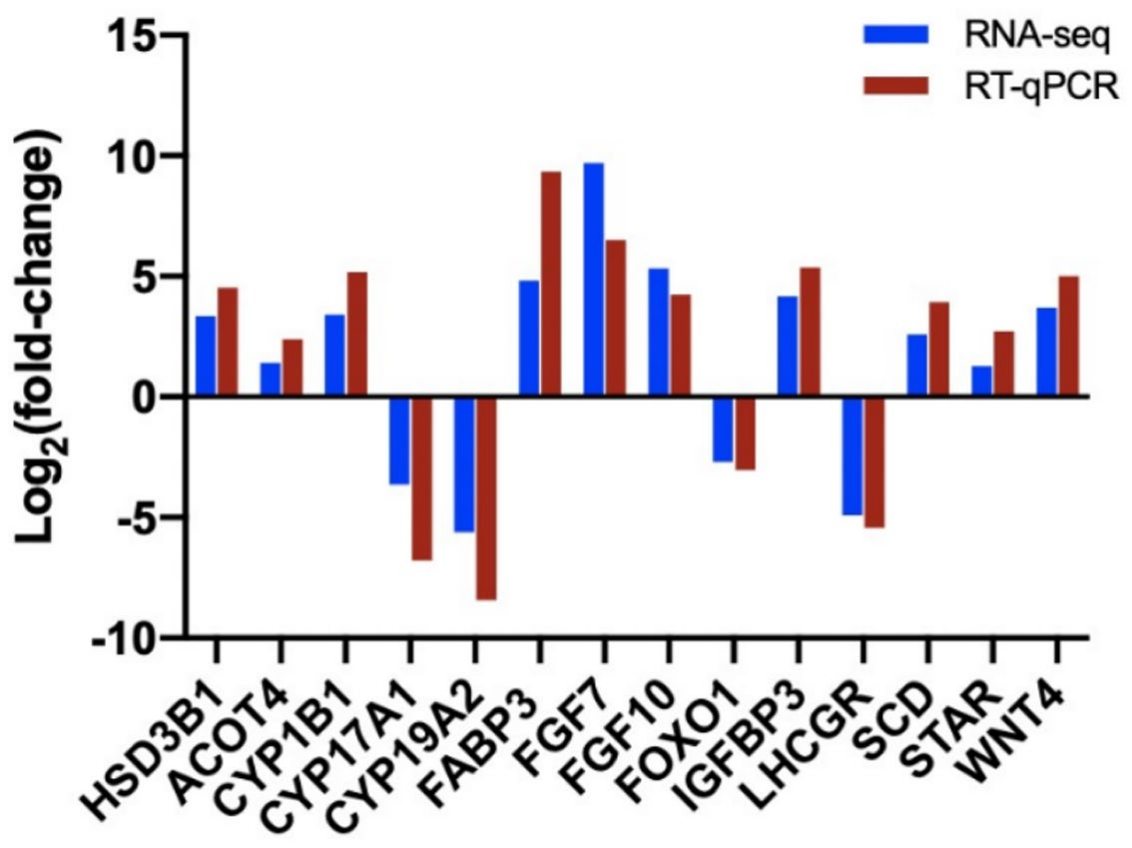
Validation of sequencing results by RT-qPCR analysis of 14 randomly selected DEGs. The blue columns refer to the RNA-seq and the red column refers to RT-qPCR.

We confirmed the fold changes of randomly selected DEGs related to follicular cysts by RT-PCR. The results confirmed the transcriptomic data (Figure 10). Three technical replicates and three biological replicates were performed for each group of samples and the relative expression level of each gene was calculated using the 2^−ΔΔCT^ method for data quantification.

### 3.8 SNPs of DEGs and their effects on gene expression

We compared transcriptomic data from follicular cysts to the reference genome and identified non-synonymous mutations in three genes: IBSP, LDHB and PRLR. These genes are involved in several pathways involved in follicular cyst formation: ECM-receptor interaction (IBSP), PI3K-Akt signaling pathway (IBSP and PRLR) and metabolic pathways (LDHB). The LDHB gene also has a mutation in the 5′-untranslated region. These data indicate that LDHB, IBSP and PRLR could be used as markers for initial detection of follicular cysts.

## 4 Discussion

High-throughput sequencing has become an effective means to explore the genes and molecular mechanisms underpinning various diseases. We used this technology to study the transcriptomic and metabolomic changes in porcine follicular cysts. After annotation, enrichment and functional classification of sequencing data, we identified a number of pathways and categories related to tumors. These predicted biological processes and pathways contribute to a better understanding of the transcriptional changes that occur during the formation of follicular cysts. These data may help address the reproductive problems caused by follicular cysts.

The transcriptomic analysis and prediction of upstream regulators in this study showed that *FGF7* and *FGF10* mRNA was significantly up-regulated in TIMG cells from follicular cysts compared with normal mature follicles. Moreover, KEGG analysis found that these two genes were enriched in the MAPK signaling pathway. FGF activates the MAPK pathway to regulate follicle development (17). Under physiological conditions, FGF7 is expressed in TIMG cells of ovarian follicles and binds to the FGFR2 receptor to activate the MAPK pathway and regulate the proliferation and differentiation of follicle cells, follicle maturation and corpus luteum formation (18, 19). However, abnormally high expression of FGF7, an important mitogen, promotes the invasion and migration of cancer cells (20), while inhibition of FGF7 signaling can reduce the migration of cancer cells (21). FGF10, another important member of the FGF family, activates the MAPK pathway by binding to FGFR1B and FGFR2B receptors (22). FGF10 is expressed at all stages of follicle development and its mRNA and proteins are localized in theca interna cells and granulosa cells (23, 24). Abnormal FGF10 expression is often closely related to tumor occurrence, and the expression of FGF10 in high-grade ovarian tumors is significantly different from that in normal tissues (25). Furthermore, FGF10 up-regulation can be used as a biomarker to predict the survival of patients with ovarian epithelial cancer (26). Although there are obvious differences between follicular cysts and tumors, we speculate that high levels of FGF7 and FGF10 expression may participate in the formation of follicular cysts in sows through activation of the MAPK signaling pathway.

Lactate dehydrogenase (LDH), which catalyzes the conversion of pyruvate to lactic acid, is one of the main glycolytic enzymes (27). Increased LDHB expression indicates up-regulation of glycolysis, which is closely related to activation of primary follicles (28). LDH is also used as a marker for ovarian tumors (29); serum LDH is significantly higher in patients with malignant ovarian cancer than in patients with benign ovarian tumors (30). In our study, the LDHB gene was significantly up-regulated in the TIMG cells of follicular cysts, and there was a mutation in the 5′-untranslated region of LDHB, indicating that increased LDH correlated with the occurrence of follicular cysts.

Glucocorticoids play an important role in mammalian physiological processes, such as stress, immune regulation, energy metabolism and homeostasis maintenance (31–34). Cortisol is the most important glucocorticoid and is an important indicator reflecting stress levels (35). The combination of cortisol and follicle cortisol-binding protein regulates follicle development in the ovary (36). Stress-mediated activation of the hypothalamic–pituitary–adrenal axis stimulates glucocorticoid production (37) and the blood cortisol concentration of stressed sows remains at a high level before follicular cyst formation, while the blood cortisol concentration of sows under stress after cyst formation or without cyst formation is normal (38). Furthermore, elevated cortisol affects aromatase synthesis, follicular development and estrus (39–41). In this study, through transcriptomic analysis of TIMG cells, we found that StAR and CYP11A1, as well as CYP17A1 and 3β-HSD were significantly enriched in the signaling pathway of cortisol synthesis and secretion in follicular cysts. These enzymes not only participate in follicle development, but are also key components of the cortisol synthesis pathway. The enrichment of this pathway further confirms the key role of cortisol in the development of follicular cysts.

Ovulation requires rupture of the follicular membrane. Polyunsaturated fatty acids are important components of cell membrane phospholipids, affect the stability of the cell membrane, and play an important role in cell surface signal transduction. Polyunsaturated fatty acids, such as linolenic acid and docosahexaenoic acid (DHA), are important components of all cell and mitochondrial membranes (42, 43), and have a direct impact on the catalytic reactions of membrane enzymes (44), receptor activity (45), transmembrane operation, metabolic rate and other functions (46). *In vivo*, linolenic acid can not only inhibit the proliferation of prostate cancer, breast cancer and bladder cancer cells (47–49), but can also inhibit the proliferation, adhesion and invasion of colon cancer cells (50). However, a large intake of linolenic acid can increase the risk of ovarian tumor (51). Similar to linolenic acid, DHA is a long-chain polyunsaturated fatty acid that can stimulate granulosa cell proliferation and steroid production (52), and promote follicle maturation and ovulation (53, 54). Treatment of lung cancer cell lines *in vitro* with different concentrations of DHA can inhibit cancer cell proliferation and promote apoptosis (55, 56). Furthermore, a study using scattering microscopy showed that polyunsaturated fatty acid metabolism is dysregulated in the ovarian tissue of a rat model of polycystic ovary syndrome (57), while increased polyunsaturated fatty acid levels have been observed in ovarian cancer stem cells, which supports the growth and migration of cancer cells (58). Furthermore, the mRNA of acyl-coenzyme A thioesterase, which controls DHA synthesis, was significantly increased and the concentration of DHA was increased in the metabolites from follicular cysts cells. In our study, both the metabolome and transcriptome of follicles were enriched in the biosynthesis of unsaturated fatty acids. Although follicular cysts and tumors have distinct features, we speculate that this increase in unsaturated fatty acids may reflect disturbed fatty acid metabolism within follicular cell membranes, interfere with normal follicle development and promote cyst formation.

Dysregulated sphingolipid metabolism is another key mechanism in ovarian cancer (59). Sphingomyelin signaling pathways introduce extracellular signaling molecules into cells through the cell membrane to exert effects on cell growth, proliferation, apoptosis and differentiation (60, 61). Sphingosinol and S1P are important molecules in sphingomyelin signaling pathways (62). These molecules are continuously synthesized and catabolized by enzymes to achieve a dynamic balance, thereby regulating biochemical reactions and maintaining the normal physiological functions. In recent years, studies have shown that sphingomyelin signaling pathways are closely related to a variety of diseases, such as heart failure, hypertension (63), diabetes (64), fatty liver and Alzheimer’s disease (65, 66). Our study shows for the first time that, compared with normal follicles, S1P mRNA is significantly up-regulated, and the metabolite sphingosine is significantly increased, in TIMG cells of follicular cysts. These results indicate that dysregulated sphingomyelin signaling plays an important role in follicular cyst formation.

## 5 Conclusion

The transcriptomic and metabolomic analysis of theca interna cells of follicular cysts showed significant enrichment of genes related to cancer, cortisol synthesis and secretion, and significant changes in the unsaturated fatty acid synthesis pathway and lipid metabolism, which may have disturbed follicular homeostasis. These results indicate a strong correlation between the formation of follicular cysts and tumors. This study lays a foundation for further research into the mechanisms of follicular cyst formation.

## Data availability statement

Original datasets are available in a publicly accessible repository and can be found in the NCBI database under accession number: PRJNA1033441.

## Ethics statement

The animal studies were approved by Institute of Animal Husbandry and Veterinary Medicine, Beijing Academy of Agriculture and Forestry Sciences, Beijing 100,097, China. The studies were conducted in accordance with the local legislation and institutional requirements. Written informed consent was obtained from the owners for the participation of their animals in this study.

## Author contributions

MP: Writing – original draft, Writing – review & editing, Formal analysis. JD: Writing – original draft, Writing – review & editing, Formal analysis. JC: Data curation, Writing – review & editing. YL: Data curation, Project administration, Writing – review & editing. YQ: Funding acquisition, Project administration, Writing – review & editing.

## Footnotes

^1^https://submit.ncbi.nlm.nih.gov/subs/sra/

^2^https://www.mzcloud.org/

^3^http://www.nist.gov/index.html

^4^http://www.ebi.ac.uk/chebi/init.do

^5^http://www.genome.jp/kegg/

^6^http://www.metaboanalyst.ca/MetaboAnalyst/

^7^https://db.cngb.org/cnsa/
