# Supplementary material for: Integrated transcriptomic and metabolomic investigation of the genes and metabolites involved in swine follicular cyst formation
Source: Front Vet Sci. 2024 Jan 11;10:1298132. doi: 10.3389/fvets.2023.1298132 (PMC10808629; doi:10.3389/fvets.2023.1298132)
Supplement: Supplementary file 2 [file Table_2.DOCX]

| The top 50 pathways of theca interna follicular cysts | |
| --- | --- |
| No. | Pathway |
| 1 | Complement and coagulation cascades |
| 2 | Hematopoietic cell lineage |
| 3 | MAPK signaling pathway |
| 4 | Cortisol synthesis and secretion |
| 5 | Axon guidance |
| 6 | Osteoclast differentiation |
| 7 | Cell adhesion molecules (CAMs) |
| 8 | Cytokine-cytokine receptor interaction |
| 9 | Pathways in cancer |
| 10 | ECM-receptor interaction |
| 11 | Metabolic pathways |
| 12 | Dilated cardiomyopathy (DCM) |
| 13 | Viral protein interaction with cytokine and cytokine receptor |
| 14 | Proteoglycans in cancer |
| 15 | PI3K-Akt signaling pathway |
| 16 | Neuroactive ligand-receptor interaction |
| 17 | Pertussis |
| 18 | Calcium signaling pathway |
| 19 | Cushing syndrome |
| 20 | Ovarian Steroidogenesis |
| 21 | Rap1 signaling pathway |
| 22 | Hypertrophic cardiomyopathy (HCM) |
| 23 | Arrhythmogenic right ventricular cardiomyopathy (ARVC) |
| 24 | Phagosome |
| 25 | Staphylococcus aureus infection |
| 26 | AGE-RAGE signaling pathway in diabetic complications |
| 27 | Steroid biosynthesis |
| 28 | Chemokine signaling pathway |
| 29 | Hippo signaling pathway |
| 30 | Amoebiasis |
| 31 | Fc gamma R-mediated phagocytosis |
| 32 | Renin secretion |
| 33 | Toxoplasmosis |
| 34 | Inflammatiory bowel disease (IBD) |
| 35 | Focal adhesion |
| 36 | Malaria |
| 37 | Rheumatoid arthritis |
| 38 | Glutamatergic synapse |
| 39 | Cholinergic synapse |
| 40 | B cell receptor signaling pathway |
| 41 | Phospholipase D signaling pathway |
| 42 | Th1 and Th2 cell differentiation |
| 43 | Aldosterone synthesis and secretion |
| 44 | Steroid hormone biosynthesis |
| 45 | Leishmaniasis |
| 46 | Adrenergic signaling in cardiomyocytes |
| 47 | Wnt signaling pathway |
| 48 | Inflammatory mediator regulation of TRP channels |
| 49 | Oxytocin signaling pathway |
| 50 | Platelet activation |
